# Enhanced CO_2_ sequestration and paramylon production in acid-tolerant *Euglena gracilis*: Growth optimization and metabolic response under varying CO_2_ concentrations

**DOI:** 10.1016/j.btre.2025.e00935

**Published:** 2025-10-30

**Authors:** Tia Erfianti, Brilian Ryan Sadewo, Nugroho Dewayanto, Adib Fakhruddin Yusuf, Dedy Kurnianto, Deviko Mardyansah, Ikhnu Prawestri Wardani, Budi Setiadi Daryono, Eko Agus Suyono, Marcos, Arief Budiman

**Affiliations:** a Faculty of Biology, Universitas Gadjah Mada, Yogyakarta 55281, Indonesia; b National Research and Innovation Agency, Research Center for Food Technology and Processing, Gunungkidul 55861, Indonesia; c Department of Chemical Engineering, Faculty of Engineering, Universitas Gadjah Mada, Yogyakarta 55284, Indonesia; d Center of Excellent for Microalgae Biorefinery, Universitas Gadjah Mada, Sleman 55281, Yogyakarta, Indonesia; e Department of Civil and Environmental Engineering, Universitas Gadjah Mada, Jl. Grafika Kampus No.2, Senolowo, Sinduadi, Mlati, Sleman, Yogyakarta 55284, Indonesia; f Faculty of Forestry, Universitas Gadjah Mada, Jl. Agro Bulaksumur No.1, Caturtunggal, Yogyakarta, Kabupaten Sleman, Daerah Istimewa Yogyakarta 55281, Indonesia; g Master Program in System Engineering, Universitas Gadjah Mada, Yogyakarta 55281, Indonesia; h School of Mechanical and Aerospace Engineering, Nanyang Technological University, 50 Nanyang Avenue, Singapore 639798, Singapore

**Keywords:** Carbon fixation, Euglena gracilis, Fatty acids, Paramylon

## Abstract

*Euglena gracilis* is a microalgae suited for acidic environments and efficient CO₂ absorption, making it a strong candidate for biological carbon fixation. This study examined its growth, biomass, lipid and paramylon production, and metabolic responses under varying CO₂ levels (0.04 %, 5 %, 15 %, 30 %). Optimal results were observed at 5 % CO₂, with peak biomass (0.407 ± 0.099 g/L), lipid (0.189 ± 0.025 g/L), and paramylon (0.0159 ± 0.0034 g/L) on day 6. SEM showed oval to ellipsoid paramylon granules (0.5–2.5 µm), while GC–MS revealed isophytol acetate (31.44 %) as the major fatty acid. Upregulation of *RubisCO* and *EgGSL1* genes indicated enhanced carbon fixation and paramylon synthesis. A 12 L pilot-scale culture confirmed high CO_2_ uptake (0.429 g·L⁻¹·day⁻¹). These findings support *E. gracilis* as a promising platform for CO_2_ sequestration, bioresource production, and paramylon-based bioproducts, with further research needed for industrial scalability.

## Highlights

• *Euglena gracilis* strain was isolated from the highlands of Dieng, Indonesia.
• CO_2_ concentration significantly influenced biomass and metabolite production.
• Optimal conditions enhanced lipid, protein, and paramylon yields.
• Findings support large-scale cultivation for biofuel and pharmaceutical uses.

## 1 Introduction

The global trend of rising CO_2_ levels is continuing. In 2024, the atmospheric concentration of carbon dioxide (CO_2_) rose to 425.4 ppm (ppm), up from 419.3 ppm in 2023, marking a 51 % increase compared to preindustrial levels [1]. Mondal et al. (2017) reported that approximately five gigatons of carbon dioxide are emitted annually via power plants, transportation, industrial plants, and cement production [2]. Increasing CO_2_ levels on Earth results in rising sea levels, heightened greenhouse gas concentrations in the atmosphere, more frequent and prolonged heat waves, melting ice sheets in Greenland and Antarctica, and loss of biodiversity [3]. The depletion of the ozone layer and the rise in heat waves worsen the adverse effects, resulting in an increase in heat-related illnesses and mortality, which are the most serious consequences of the increase in CO_2_ levels in the environment [4,5].

Many methods for capturing and converting carbon have been suggested to address the issue of CO_2_ emissions. CO_2_ can be reduced from flue gas by solvent absorption, physical adsorption, membrane separation, or cryogenic distillation [6]. CO_2_ can be captured from the exhaust, reformed gasses, or power plants using the following primary methods: pre-combustion capture, post-combustion capture, and oxy-fuel combustion [7]. Nowadays, carbon dioxide is removed from flue gas by liquid absorption with solvents, including selexol, rectisol, and mono-ethanol-amine. However, these processes are resource-intensive in terms of both capital and energy requirements. An inexpensive and simple method for regenerating is through physical adsorption using a solid adsorbent, which can selectively reduce CO_2_ from a mixture of exhaust gas [8]. In addition to these physico-chemical methods, biological approaches, such as the use of microalgae, have been suggested as a potentially sustainable and eco-friendly option for CO_2_ mitigation [7].

Microalgae have greater growth rates, photosynthetic efficiency, and environmental adaptability compared to higher plants [9]. Microalgal cells consist of 50 % carbon and can produce 1 kg of biomass by sequestering 1.8 kg of CO_2_. The resulting biomass has potential applications in various fields such as food, feed, and bioenergy; however, its industrial feasibility depends on further techno-economic assessment and process optimization. Microalgal carbon sequestration is regarded as one of the most beneficial to the environment, with safe and sustainable technology for carbon capture and utilization (CCU) [10]. Flue gas from coal-fired power stations contains approximately 10–25 % CO_2_, it is significantly greater than the ambient CO_2_ concentration of 0.04 % [11,12].

Microalgal growth and the efficiency of CO_2_ fixation can be influenced by many cultivation conditions, such as temperature, light, pH, CO_2_ mass transfer, and nutrient availability in the cultural medium [13]. Furthermore, the injection of CO₂ into the culture medium directly leads to acidification [14]. Therefore, selecting microalgal strains with acid tolerance was essential for this study. The genus *Euglena* is one potential strain with this capability. In our initial research, we successfully identified *E. gracilis* from the Dieng plateau in Wonosobo, Central Java, Indonesia [15,16]. We observed that the isolated *E. gracilis* was able to grow in acidic environments with a pH between 2.5 and 3.5.

*Euglena* possesses various enzymes involved in the carbon fixation pathway, such as carbonic anhydrase and ribulose-1,5-bisphosphate carboxylase (RubisCo). Carbonic anhydrase (*CA*) catalyses the conversion of CO_2_ into bicarbonate (HCO_3_⁻) [17]. The genes encoding *CA* include *α-CA, β-CA, γ-CA, and θ-CA,* while RubisCo catalyzes the reaction between CO_2_ and ribulose-1,5-bisphosphate (RuBP), with its corresponding genes being *rbcL* and *rbcS* [18]. The expression of these genes involved in the carbon fixation pathway is regulated by CO_2_ concentrations.

In addition, *E. gracilis* also contains paramylon, a storage polysaccharide composed of β−1,3-glucan. This compound has a crystalline structure and is formed as granules within the cell [19]. Paramylon has been extensively studied and utilized due to its potential applications in the fields of pharmaceuticals, nutraceuticals, and agriculture. This polysaccharide is a linear β−1,3-glucan resembling curdlan and possesses a crystalline structure stabilized by intramolecular hydrogen bonds [20]. The synthesis of paramylon, a β−1,3-glucan storage polysaccharide in *E. gracilis,* is mediated by specific genes known as Glucan Synthase-Like genes, among which *EgGSL1* (*Euglena gracilis* Glucan Synthase-Like 1) plays a crucial role. *EgGSL1* encodes a β−1,3-glucan synthase enzyme responsible for catalyzing the polymerization of glucose residues via β−1,3-glycosidic linkages, leading to the formation of linear paramylon chains [21].

This study aims to evaluate the ability of *E. gracilis* cultivated under different CO_2_ concentrations, including; ambient CO_2_ (0.04 %) as control, 5 %, 15 %, and 30 %. In this study, we also assessed the effect of various CO_2_ stresses on the growth profile, biomass content, lipids, growth kinetics, and fatty acid compounds contained in *E. gracilis*. In this study, we also validated at the mRNA transcript level using a qRT-PCR approach to analyze the expression levels of key carbon fixation genes (*rbcL* and *CA*), as well as the *EgGSL1* gene, which plays a role in paramylon (β−1,3-glucan) synthesis. Lastly, we examined the CO_2_ absorption capability of *E. gracilis* to assess its potential for CO_2_ sequestration at the laboratory scale, which may serve as a foundation for future research on industrial applications, pending a comprehensive techno-economic evaluation.

## 2 Materials and methods

### 2.1 Cultivation of *E. gracilis* under various CO_2_ concentrations

Cultivation of *E. gracilis* species sourced from the laboratory of Biotechnology, Faculty of Biology, Universitas Gadjah Mada, Yogyakarta, Indonesia, was carried out with a Cramer Myers (CM) medium pH 3.5 [22]. Table 1. shows the compositions of the CM medium that we used in this study.

**Table 1:** The components of the CM medium used in this study.

**Compositions**	**Concentrations (mg/L)**
(NH_4_)_2_SO_4_	1.000
KH_2_PO_4_	1.000
MgSO_4_ .7H_2_O	200
CaCl_2_·2H_2_O	20
Fe_2_(SO_4_)_3_. 7H_2_O*	3.0
MnCl_2_. 7H_2_O*	1.8
CoSO_4_. 7H_2_O*	1.5
ZnSO4. 7H2O*	0.4
CuSO_4_. 5H_2_O*	0.02
Na_2_MoO_4_. 2H_2_O	0.2
Vitamin B_1_	0.1
Vitamin B_12_	0.0005

Note: Compositions marked with * are trace metal components.

**Fig. 1:**
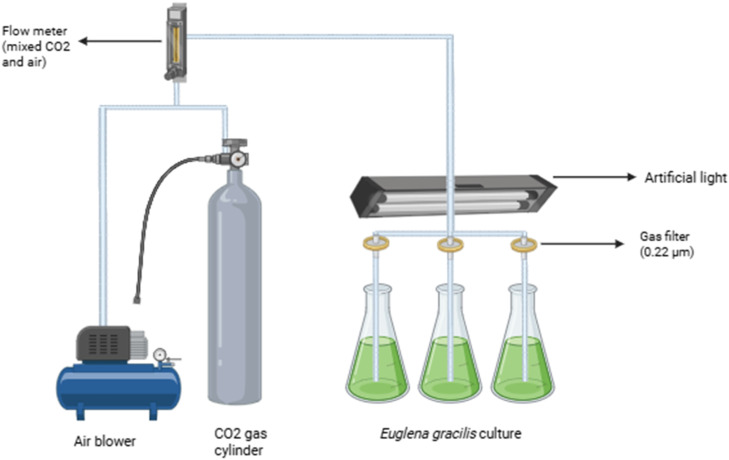
Schematic diagram of the experimental set-up.

Each microalgae was initially cultivated in a total volume of 500 mL CM medium, aerated with various CO_2_ concentrations ranging from 0.04 % (control), 5 %, 15 %, and 30 %. The illumination intensity was 3100 lux, and both control and treatment groups were provided with the same light intensity. The photoperiod used in this study was 24:0 (continuous light) with a cultivation room temperature of 20 °C. Lab-scale cultivation was conducted for 12 days until microalgae entered the death phase. The schematic diagram of this study is presented in Fig. 1 below.

### 2.2 Cell density measurement

Daily cell counts were conducted to create a growth curve for *E. gracilis*. Following that, cell density was determined using a Neubauer Haemocytometer from China. Microalgal cells were examined using an Inverted Microscope (BDS400 DRAWELL, China) at a magnification of 10×.

### 2.3 Determination of dry weight

The biomass of *E. gracilis* was measured by GF/C filter paper (No. 1822–047, China) with 10 mL of culture. Each measurement was performed in triplicate for biological replicates (*n* = 3 per time point). Prior to use, the filter papers were pre-dried in an oven at 100 °C to ensure accurate biomass measurement. Biomass measurements were conducted every three days. Dewatering of the *E. gracilis* culture was achieved by using vacuum filtration, followed by drying the filter papers in a vacuum oven. This process helps to remove excess moisture from the biomass, ensuring accurate dry weight measurements for the subsequent Ash-Free Dry Weight (AFDW) determination. Besides, the filter papers were dried using an oven at 100 °C for 1–2 h. The sample of *E. gracilis* culture was then centrifuged at 4000 rpm for 5 min to separate the pellet (biomass) from the supernatant (medium). The biomass accumulated on the filter paper was subsequently measured using an analytical balance. Biomass determination was carried out using the AFDWmethod, which represents the true organic biomass by subtracting the ash (inorganic) content from the total dry weight. The dry biomass was calculated using the following formula:

(1) $$\text{Biomass}\;\left(\frac{\text{mg}}{\text{mL}}\right)=\frac{\left(W1\right)-\left(W0\right)}{\left(\;\text{Volume}\;\text{of}\;\text{sample}\right)}$$

W1= Final weight of the filter paper after drying (mg)

W0= Initial dry weight of filter paper (mg)

In addition, biomass productivity, specific growth rate, division per day (Dd), and doubling time (Td) was calculated using the following formula according to Gani et al. (2016);

(2) $$\text{Specific}\;\text{growth}\;\text{rate}\;\left(\mu /\text{day}\right)=\frac{\ln\left(\text{Nf}/\text{Ni}\right)}{\text{Tf}-\text{Ti}}$$

(3) $$\begin{array}{c} \text{Division}\;\text{per}\;\text{day}\;\left(\text{Dd}\right)=\frac{\mu }{\ln2} \end{array}$$

(4) $$\text{Doubling}\;\text{time}\;\left(\text{Td}\right)=\frac{1}{\text{Dd}}$$

(5) $$\text{Biomass}\;\text{productivity}=\frac{\text{Nf}-\text{Ni}}{\text{Tf}-\text{Ti}}$$

Nf and Ni were defined as the biomass content measured at time Tf and Ti, respectively [23,24].

### 2.4 Determination of lipid content

Lipid content analysis was conducted using the Bligh and Dyer (1959) method [25]. An algal culture sample was put into a 15 mL conical tube and then extracted to the maximum volume of 15 mL. The lipid solution was measured after completing overnight drying. Lipid content was calculated using the following formula:

(6) $$\text{Lipid}\;\text{content}\;\left(\frac{\text{mg}}{\text{mL}}\right)=\frac{D_{t}-D_{0}}{V}$$

D_t_: The weight of the aluminium foil and sample (lipid) after drying (mg).

D_0_: Initial weight of the aluminium foil (mg)

V: Sample volume (mL)

### 2.5 GC–MS analysis

100 µL of pyridine was added to a one-milliliter pellet that was obtained from 2 L of culture. The sample was centrifuged at 4 min, 4000 rpm, and 4 °C. Sonication was then performed to break the samples. About 99 µL of N,O-Bis(trimethylsilyl)trifluoroacetamide (BSTFA) and 1 µL of chlorotrimethylsilane (TMCS) were added as derivatization reagents, followed by additional sonication. Sonication was then performed to break the samples, and repeated after adding the derivatization reagents (BSTFA and TMCS) to ensure complete reaction. The extract was then dried for 30 min at 37 °C in an oven and subsequently reconstituted with 1 mL of n-hexane as a solvent. After filtration, the dissolved extract was injected into GC–MS (QP2010, Shimadzu, Japan).

The GC condition was as follows: a single capillary column (30 *m* × 0.25 mm I.D., 0.25 µm film thickness) with a split ratio of 1:0, an injection temperature of 290 °C, and a column pressure of 108.1 kPa. The programmed temperature started at 70 °C, increasing by 6 °C per minute, and reached a final temperature of 285 °C, which was held constant. Detected organic compounds were identified using the NIST II library database, and the relative content of each compound was quantified based on the percentage area of its GC–MS peak. The composition and concentrations of the FAME standard are presented in Supplementary Table S1.

### 2.6 Gene expression using qRT-PCR

Gene expression analysis started from isolating *E. gracilis* RNA in the late exponent phase using an RNA isolation kit (Zymo research Quick-RNA Plant Miniprep Kit, USA), following the protocol provided. Next, RNA was converted into cDNA using Toyobo ReverTra Ace® qPCR RT Master Mix with gDNA remover, Japan, following the attached protocol. The next step was qRT-PCR analysis using Bioline SensiFAST SYBR No-ROX Kit, USA, according to the procedure. The qPCR stage was performed using the CFX96 Touch Real-Time PCR Detection System instrument, USA with the following PCR cycle. Table 2 represents the qPCR cycle and stage, while Table 3 shows the primers used in this study.

**Table 2:** qPCR cycle and stage in this study.

**Stage**	**Temperature**	**Time**	**Cycles**
Pre-denaturation	95 ºC	2 min	1x
Denaturation	95 ºC	5 s	40x
Annealing	55 ºC	10 s	
Extension	72 ºC	20 s

**Table 3:** List of primers used in this study.

Gene	Forward (5′−3′)	Reverse (3′−5′)	References
*RubisCO*	CTCCACCCGCTCCGTCTAAG	GACAAACTCGTGCGACATTCTT	[26]
*CA*	TGTGAGCGGACAGCAACCA	GGGACGAAGAGGAGAAGAGGG	[26]
*EgGSL1*	AGCGCATCCCGGTCACAATG	TTGTCCCACGTGTACGCCAC	[27]
*Ef1α (reference)*	CGAAGAACGACCCTGCAAAG	CTGTCCAGGATGGTTCAGAATG	(GenBank; ACO50110)

### 2.7 Paramylon measurement and observation

A 20 mL sample was mixed with 4 mL of acetone [28], centrifuged at 4000 rpm for 10 min, and the resulting pellet was resuspended in 3 mL of 1 % SDS and 5 % Na₂EDTA. After heating at 95 °C for 30 min, the sample was centrifuged and purified three times with 1 mL of ddH₂O. The pellet was then dissolved in 2 mL of 0.5 N NaOH. A 500 µL aliquot was used to assess paramylon content following [29], with absorbance recorded at 480 nm. Paramylon content was quantified using a standard linear regression equation derived from β−1,3-glucan standards. The extracted paramylon granules were examined using scanning electron microscopy (SEM).

### 2.8 Evaluation of CO_2_ biofixation ability

To evaluate the CO₂ capture efficiency of microalgae, a systematic approach was employed, incorporating gas flow measurements, stoichiometric calculations, and normalization to cultivation volume. The methodology was designed to quantify the amount of CO₂ sequestered by microalgae over time under controlled cultivation conditions.

The experimental setup involved introducing a gas mixture containing a known concentration of CO₂ into the microalgae culture system. The CO₂ gas concentration (ppm), CO₂ flow rate (L/min), and airflow rate (L/min) were carefully controlled to maintain a consistent gas environment. The proportion of CO₂ in the gas flow was set at 5 % (50,000 ppm). The system operated under ambient pressure conditions, with total pressure assumed to be 1 atm, and gas flow was maintained at 2.1 L/min throughout the experiment.

To determine the extent of CO₂ uptake by microalgae, inlet and outlet CO₂ concentrations were continuously measured. The CO₂ concentration in the inlet gas was recorded ppm, while the outlet CO₂ concentration was measured after interaction with the microalgae culture. The CO₂ reduction, calculated as the difference between inlet and outlet concentrations, corresponding in percentage unit.

(7) $$\%\;CO_{2}\;\text{reduction}=\;\frac{CO_{2\;\text{inlet}\;\text{concentration}}-\;CO_{2\;\text{outlet}\;\text{concentration}}}{CO_{2\;\text{inlet}\;\text{concentration}}}$$

To quantify CO₂ capture in molar terms, the ideal gas law was applied, incorporating parameters such as total gas flow, temperature, and pressure. Given the temperature of 27 °C (300 K) and an ideal gas constant (0.082 Latm/mol.K), the total molar flow of CO₂ at the inlet was calculated to be moles per minute. Subsequently, the molar amount of CO₂ captured was derived based on the observed reduction in CO₂ concentration, yielding a capture rate in moles per minute unit.

(8) $$\text{Mole}\;\text{total}\;\left(\text{Air}+CO_{2}\right)=\;\frac{\text{Total}\;\text{flow}\;\text{rate}\left(\frac{L}{\text{min}}\right)x\;\text{Pressure}\;\left(\text{atm}\right)}{R\;\left(\frac{L\;\text{atm}}{\text{mol}\;K}\right)x\;\text{Temperature}\;\left(K\right)}$$

where R is the ideal gas constant, equal to 0.0821 L·atm/mol·K.

Then, the amount of CO₂ captured was calculated by determining the difference between the inlet and outlet moles of CO₂:

(9) $$\text{Mole}\;CO_{2}=\%\;CO_{2\;}x\;\text{Mole}\;\text{total}$$

(10) $$\text{Mole}\;CO_{2\;\text{captured}}=\;\text{Mole}\;CO_{2\;\text{inlet}\;}-\;\text{Mole}\;CO_{2\;\text{outlet}\;}$$

The next step involved converting the molar amount of captured CO₂ into mass-based units using the molecular weight of CO₂ (44 g/mole). This calculation resulted in a CO₂ capture rate in g/min unit, which was further extrapolated to obtain the total CO₂ captured per day, yielding a value in mass unit g/day or kg/day.

(11) $$CO_{2\;\text{captured}\;\text{rate}}\;\left(\frac{\text{gram}}{\text{day}}\right)=\;\text{Mole}\;CO_{2\;\text{captured}}\;x\;44\;\left(\frac{\text{gram}}{\text{mole}}\right)\;x\;60\frac{\text{min}}{\text{hour}}\;x\;24\;\frac{\text{hour}}{\text{day}}$$

To normalize the CO₂ capture efficiency relative to the culture volume, the total cultivation volume of 12 liters was considered. By dividing the total CO₂ captured per day (in grams) by the culture volume, the CO₂ capture rate per liter of algae was determined to be in g/L/day unit. This value provides a standardized measure of carbon sequestration efficiency, allowing for comparative analysis across different experimental conditions.

(12) $$CO_{2\;\text{captured}\;\text{rate}\;\text{per}\;\text{volume}}\;\left(\frac{\text{gram}}{L\;\text{day}}\right)=\frac{CO_{2\;\text{captured}\;\text{rate}}\;\left(\frac{\text{gram}}{\text{day}}\right)}{\text{Cultivation}\;\text{Volume}\;\left(L\right)}$$

### 2.9 Statistical analysis

The growth rate, biomass, lipid, and paramylon content of *E. gracilis* were analyzed using ANOVA, and mean differences were analyzed using the Duncan Multiple Range Test (DMRT) using IBM's Statistical Product and Service Solutions (SPSS) software (Version 26, IBM Corporation, USA). The significance of the data was determined by a p-value of 0.05 at a significance level of 5 %. All data were represented using Origin software (Version 2023).

## 3 Results and discussion

### 3.1 The growth of *E. gracilis* under various CO_2_ concentrations

The highest cell density was recorded on day 6, and the optimal growth trajectory was achieved under 5 % CO₂ treatment. *E. gracilis* entered a latency phase from day 0 to day 1, which was followed by an exponential phase from day 1 to day 6. Furthermore, the control, 15 % CO_2_, and 30 % CO_2_ regimens exhibited comparable growth patterns, with the highest growth occurring on the sixth day of cultivation. On the seventh day, all treatments entered the decline phase, and all treatments except for 5 % CO_2_ transitioned to the stationary phase.

**Fig. 2:**
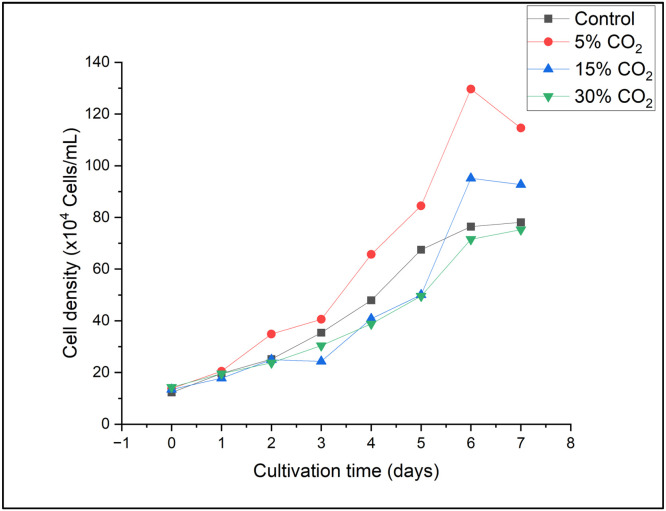
Cell density of *E. gracilis* under various CO_2_ concentrations.

Fig. 2 shows that *E. gracilis* demonstrated the highest growth rate when subjected to the 5 % CO_2_ treatment relative to the other treatments. According to Yuan et al. (2024), *E. gracilis* demonstrated a cell density that was 1.79 times higher when exposed to a CO_2_ concentration of 5 %, in contrast to both the control and a CO_2_ concentration of 20 %. The results indicate that *E. gracilis* exhibits considerable tolerance to CO_2_ levels of up to 30 % and possibly beyond; nonetheless, these elevated concentrations adversely affect the growth of *E. gracilis*, as the strain must adjust to a CO_2_-rich environment. This study indicates that *E. gracilis* requires adaptation to survive high CO_2_ concentrations. Thus, the evolution of adaptation on a laboratory scale needs to be evaluated. Previous research also reported that adaptive evolution serves as a powerful approach for identifying algal strains that excel in CO_2_ capture [30]. Our observations suggest that *E. gracilis* cultivated under the 30 % CO₂ treatment in this study either could not acclimate to the elevated CO_2_ stress in the culture medium or underwent shock, as evidenced by its growth pattern, which resembled that of the control group. Further evidence confirming this finding was provided by a previous study conducted by Guo et al. (2017). The research demonstrated that the proliferation of cells can be blocked by elevated CO_2_ levels, which results in a greater emphasis on catabolic processes than on anabolic processes. In certain situations, the proliferation of microalgae can be inhibited by excessive CO_2_ levels, although the optimal amount of CO_2_ promotes their growth [31].

### 3.2 Biomass content of *E. gracilis*

**Fig. 3:**
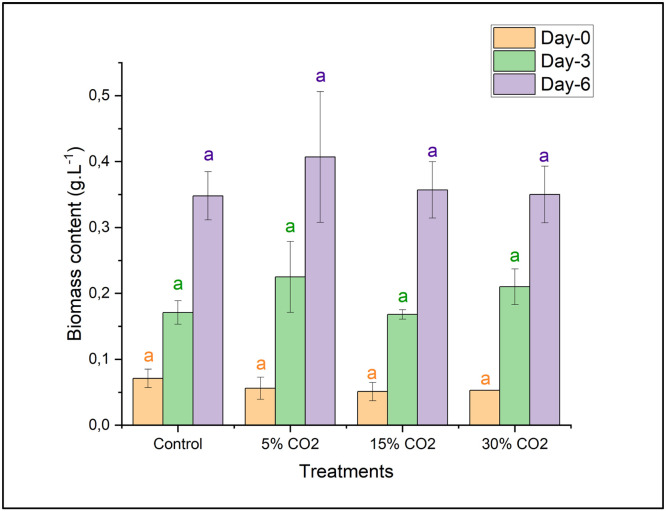
Biomass content of *E. gracilis* under various CO_2_ concentrations. Different letters (a, b) indicate statistically significant differences among treatments based on Duncan’s multiple range test (*p* < 0.05).

According to Fig. 3, the dry biomass obtained on days 0, 3, and 6 of cultivation showed no statistically significant differences, as determined by ANOVA (*p* ≤ 0.05) followed by DMRT post-hoc analysis. However, the highest dry biomass content was observed on days 3 and 6 in *E. gracilis* cultivated with the addition of 5 % CO₂, reaching 0.200 ± 0.053 g/L and 0.407 ± 0.099 g/L, respectively. On day 0 of cultivation, there was no difference in initial biomass between all treatments, indicating that the initial inoculum was equal. Furthermore, although there were visual variations in biomass between treatments on days 3 and 6 of cultivation, the differences were not statistically significant. This indicates that the variation in CO₂ concentration (5 %, 15 %, and 30 %) did not have a significant effect on increasing the biomass of *E. gracilis* compared to the control.

Based on these findings, 5 % CO₂ appears to be an optimal condition for *E. gracilis* biomass production. This outcome is likely due to the absence of prior adaptive evolution, causing a significant decline in biomass when the CO₂ concentration was increased. Previous research by Xin et al. (2023) demonstrated that subjecting *E. gracilis* to CO₂ gradient domestication enhanced biomass production by up to 1.79 times compared to wild-type *E. gracilis* strains. Similarly, Chiu et al. (2008) reported that *Chlorella* sp. cultivated under 10–15 % CO₂ exhibited growth inhibition; however, this effect could be mitigated by pre-adapting the inoculum to 2 % CO₂.

Furthermore, the introduction of carbon dioxide into the culture through bubbling may result in a nutritional imbalance in the carbon-to-nitrogen (C/N) ratio, which in turn may result in a change in the composition and quality of the biomass [32,33]. In the study conducted by Farooq (2022), it was shown that the biomass of *Parachlorella kessleri* HY-6 rose to 2.0 g/L when exposed to conditions of 10 % CO_2_ and light intensities of 60 and 100 μmol m⁻² s⁻¹ simultaneously [34]. Additionally, acidification is a direct consequence of CO_2_ injection into the culture medium [14]. As a result, the utilization of microalgal strains that can tolerate acid was crucial for the current research.

### 3.3 Lipid content of *E. gracilis*

**Fig. 4:**
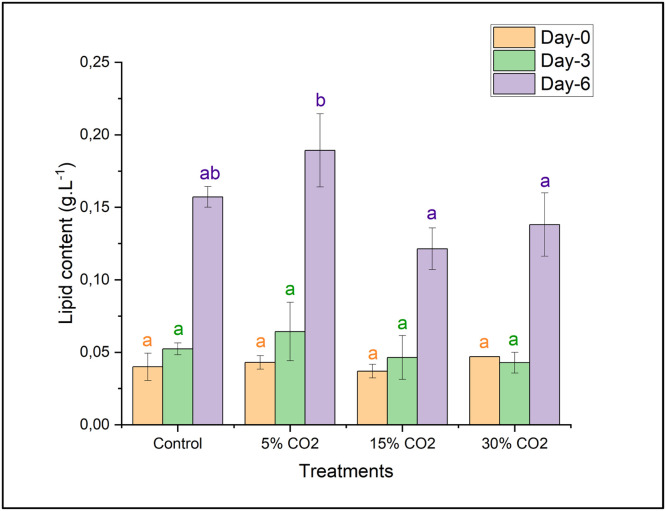
Lipid content of *E. gracilis* under various CO_2_ concentrations. Different letters (a, b) indicate statistically significant differences among treatments based on Duncan’s multiple range test (*p* < 0.05).

The lipid content of *E. gracilis* on days 0 and 3 of cultivation showed no significant differences among treatments (Fig. 4), as determined by statistical analysis using ANOVA (*p* ≤ 0.05). Further analysis using DMRT confirmed that all treatment groups belonged to the same subset, indicating no statistically significant variation. However, on day 6 of cultivation, a significant difference was observed between the 5 % CO₂ treatment and the control group compared to the other treatments. Consistent with the dry biomass results, the highest lipid content was also recorded in the 5 % CO₂ treatment, reaching 0.189 ± 0.025 g/L on day 6 of cultivation. Based on the results, it was observed that on days 0 and 3 of cultivation, lipid synthesis in *E. gracilis* did not show significant differences among treatments. However, by day 6 of cultivation, it became evident that the 5 % CO₂ treatment, followed by the control, resulted in higher lipid content compared to the 15 % and 30 % CO₂ treatments.

Our results were positively confirmed by previous research by Cheng et al. (2016) that *Chlorella* sp. under 15 vol. % CO_2_ cultivation was able to produce lipid yield reaching 1.0 g L^−1^[35]. In addition, the content of long-chain saturated fatty acids increased while short-chain saturated fatty acids decreased. Wu et al. (2016) showed that CO_2_ concentration stimulated lipid productivity and growth in *Phaeodactylum tricornutum*. Meanwhile, under high CO_2_ concentration conditions, the total percentage of unsaturated fatty acids decreased by 26.77 % and 27.93 % compared to the wild-type strain of *P. tricornutum* [36]*.*

In addition, research carried out by Wang et al. (2018) demonstrated that the total lipid content of *E. gracilis* (NIES-48) varied between 18 and 22 % of the dry biomass. An additional study that was carried out by Sun et al. (2016) demonstrated that an increase in the amounts of carbon dioxide could drive cells to move photosynthetic carbon molecules towards pathways that produce fatty acids, which would increase the creation of total triacylglycerol overall. Furthermore, it has been demonstrated that a significant amount of energy, reductants, and carbon skeleton are needed for both algal growth and lipid synthesis. In particular, a significant amount of NADPH was needed for the synthesis of lipids [37].

### 3.4 GC–MS analysis

**Fig. 5:**
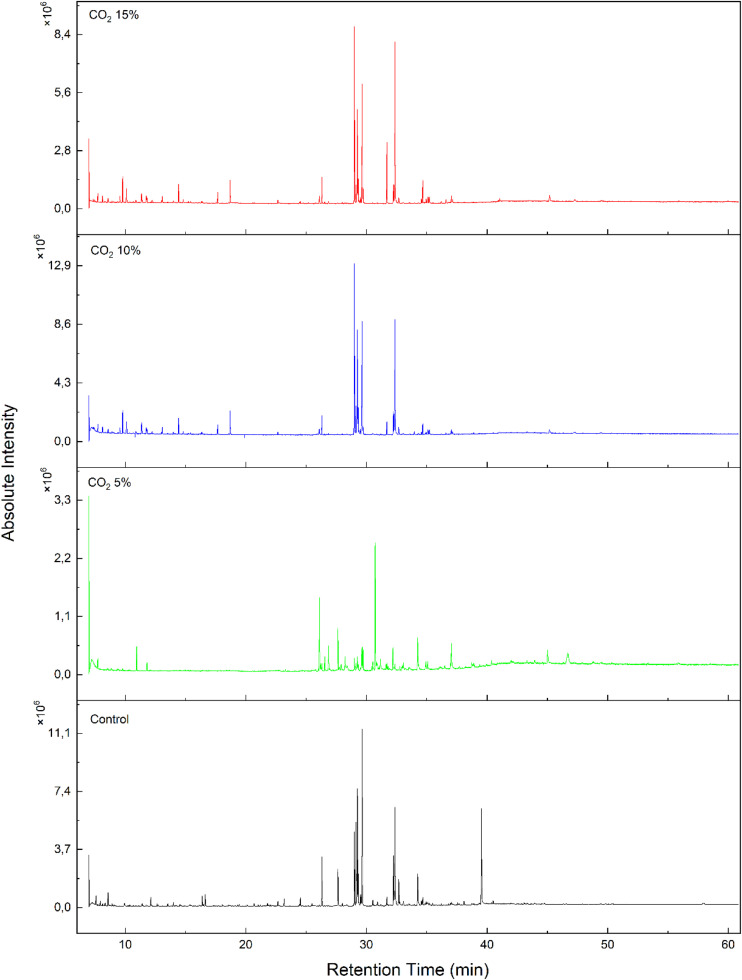
GCMS result of *E. gracilis* under different CO_2_ concentrations. The y-axis represents absolute intensity, displayed in units of ×10⁶.

Analysis of organic compounds was carried out using the GC-MS method. GCMS results are shown in Fig. 5 below.

Based on Fig. 5, the best TIC GC–MS graph was obtained in the control. In the control treatment, there were 14 organic compounds detected, including Eicosanoic acid, trimethylsilyl ester (C_23_H_48_O_2_Si), alpha.-Linolenic acid, trimethylsilyl ester (C_21_H_38_O_2_Si), and cis-5,8,11-Eicosatrienoic acid, trimethylsilyl ester (C_23_H_42_O_2_Si). Meanwhile, in 5 % CO_2_, 11 organic compounds were detected, including isophytol, acetate (C_22_H_42_O_2_), 1-Hexadecyne (C_16_H_30_), and Tridecanoic acid, 12-methyl-, methyl ester (C_15_H_30_O_2_). In 15 % CO_2_, there were 12 organic compounds detected; cis-4,7,10,13,16,19-Docosahexaenoic acid, trimethylsilyl ester (C_25_H_40_O_2_Si), cis-5,8,11-Eicosatrienoic acid, trimethylsilyl ester (C_23_H_42_O_2_Si), and Hexadecanoic acid, trimethylsilyl ester (C_19_H_40_O_2_Si). Furthermore, there were 11 organic components detected, namely; cis-5,8,11-Eicosatrienoic acid, trimethylsilyl ester (C_23_H_42_O_2_Si), 9,12-Octadecadiynoic acid, trimethylsilyl ester (C_21_H_36_O_2_Si), and Eicosanoic acid, trimethylsilyl ester (C_23_H_48_O_2_Si).

The principal component analysis (PCA) revealed clear clustering patterns that distinguished the treatment groups based on fatty acid profiles (Supplementary Figure S1). This suggests that increasing CO₂ concentrations significantly influenced the fatty acid biosynthesis pathways in *E. gracilis.* PCA revealed a clear separation of groups based on fatty acid composition, with PC1 and PC2 explaining 57.6 % and 26.4 % of the total variance, respectively (Supplementary Figure S1). This indicates that different CO₂ concentrations strongly influence fatty acid biosynthesis pathways in *E. gracilis.*

The Variable Importance in Projection (VIP) score analysis identified key compounds contributing to the separation of treatment groups (Supplementary Figure S2). Compounds with VIP scores above 1 were considered the main contributors to group differentiation, providing insight into lipid metabolism under different CO₂ treatments. Compounds with VIP scores exceeding 1 were considered the primary contributors to differentiation among treatment groups. Among them, C_10_H_26_O_2_Si_2_, C_22_H_42_O_2_, C_12_H_26_O_2_Si, and C_23_H_38_O_2_Si displayed the highest VIP scores (>1.4), indicating their strong importance in distinguishing between the treatments. According to the heatmap, these compounds showed high concentrations under the control condition, suggesting they are not strongly responsive to the CO₂ treatment. In contrast, C_17_H_36_O_2_Si, C_23_H_44_O_2_Si, and C_23_H_48_O_2_Si, all with VIP scores below 1, contributed less significantly to group separation. Furthermore, we present the list of detected organic compounds of control and treatments in Tables 4, 5, 6, 7 below.

**Table 4:** List of detected organic compounds in control.

**Ret. Time**	**Similarity Index**	**Name**	**Formula**	**Concentration (% area) ± SD**
12.125	91	Propane, 2-methyl-1,2-bis(trimethylsiloxy)-	C_10_H_26_O_2_Si_2_	0.90 ± 0.02
16.383	85	Nonanoic acid, trimethylsilyl ester	C_12_H_2_6O_2_Si	1.00 ± 0.06
24.527	91	n-Tridecanoic acid, trimethylsilyl ester	C_16_H_34_O_2_Si	1.03 ± 0.10
26.311	89	Tetra decanoic acid, trimethylsilyl ester	C_17_H_36_O_2_Si	5.80 ± 0.16
27.638	92	Hexadecenoic acid, methyl ester	C_17_H_34_O_2_	4.31 **±** 0.03
28.996	82	cis-5,8,11,14,17-Eicosapentaenoic acid, trimethylsilyl ester	C_23_H_38_O_2_Si	9.47 **±** 0.66
29.137	88	Linoleic acid trimethylsilyl ester	C_21_H_40_O_2_Si	10.75 **±** 0.70
29.252	89	alpha.-Linolenic acid, trimethylsilyl ester	C_21_H_38_O_2_Si	15.35 **±** 0.80
29.317	83	Oleic acid, trimethylsilyl ester	C_21_H_42_O_2_Si	3.71 **±** 0.26
29.644	83	Eicosanoic acid, trimethylsilyl ester	C_23_H_48_O_2_Si	18.57 **±** 4.24
32.243	89	11,14-Eicosadienoic acid, trimethylsilyl ester	C_23_H_44_O_2_Si	6.94 **±** 0.94
32.363	82	cis-5,8,11-Eicosatrienoic acid, trimethylsilyl ester	C_23_H_42_O_2_Si	15.25 **±** 1.17
32.681	84	Tetracosanoic acid, trimethylsilyl ester	C_27_H_56_O_2_Si	2.51 **±** 0.76
34.245	84	(E)-13-Docosenoic acid	C_22_H_42_O_2_	4.44 **±** 0.25

The data shows in means followed by standard deviation (*n* = 2).

**Table 5:** List of detected organic compounds in 5 % CO_2_.

**Ret. Time**	**Similarity Index**	**Name**	**Formula**	**Concentration (% area) ± SD**
26,101	90	1-Hexadecyne	C_16_H_30_	17.56 **±** 0.03
26,544	90	1-Octadecyne	C_18_H_34_	3.05 **±** 0.11
26,861	89	9-Eicosyne	C_20_H_38_	5.84 **±** 0.01
27,635	92	Tridecanoic acid, 12-methyl-, methyl ester	C_15_H_30_O_2_	9.98 **±** 0.10
28,223	91	n-Hexadecenoic acid	C_16_H_32_O_2_	4.21 **±** 0.98
29,244	87	.alpha.-Linolenic acid, trimethylsilyl ester	C_21_H_38_O_2_Si	2.30 **±** 0.66
29,711	87	Arachidonic acid	C_20_H_32_O_2_	5.18 **±** 0.38
30,726	89	Isophytol, acetate	C_22_H_42_O_2_	31.44 **±** 0.95
32,191	91	2-Methyl-1-undecanol	C_12_H_26_O	5.47 **±** 0.74
34,243	84	(E)-13-Docosenoic acid	C_22_H_42_O_2_	8.92 **±** 0.74
37,044	87	Diisooctyl phthalate	C_24_H_38_O_4_	6.07 **±** 0.06

The data shows in means followed by standard deviation (*n* = 2).

**Table 6:** List of detected organic compound in 15 % CO_2_.

**Ret. Time**	**Similarity Index**	**Name**	**Formula**	**Concentration (% area)**
26,302	89	Tetradecanoic acid, trimethylsilyl ester	C_17_H_36_O_2_Si	2.59 **±** 0.31
28,996	84	cis-4,7,10,13,16,19-Docosahexaenoic acid, trimethylsilyl ester	C_25_H_40_O_2_Si	27.17 **±** 2.03
29,127	88	Linoleic acid trimethylsilyl ester	C_21_H_40_O_2_Si	4.33 **±** 0.02
29,246	89	.alpha.-Linolenic acid, trimethylsilyl ester	C_21_H_38_O_2_Si	16.78 **±** 0.91
29,310	84	Oleic acid, trimethylsilyl ester	C_21_H_42_O_2_Si	3.49 **±** 0.21
29,633	91	Hexadecanoic acid, trimethylsilyl ester	C_19_H_40_O_2_Si	17.17 **±** 3.32
29,708	87	Arachidonic acid	C_20_H_32_O_2_	1.23 **±** 0.10
32,236	90	11,14-Eicosadienoic acid, trimethylsilyl ester	C_23_H_44_O_2_Si	3.30 **±** 0.08
32,360	82	cis-5,8,11-Eicosatrienoic acid, trimethylsilyl ester	C_23_H_42_O_2_Si	20.50 **±** 0.93
32,672	84	Eicosanoic acid, trimethylsilyl ester	C_23_H_48_O_2_Si	0.95 **±** 0.28
34,669	83	cis-7,10,13,16-Docosatetraenoic acid, trimethylsilyl ester	C_25_H_44_O_2_Si	1.84 **±** 0.16
35,105	80	cis-13,16-Docasadienoic acid, trimethylsilyl ester	C_25_H_48_O_2_Si	0.67 **±** 0.10

The data shows in means followed by standard deviation (*n* = 2).

**Table 7:** List of detected organic compound in 30 % CO_2_.

**Ret. Time**	**Similarity Index**	**Name**	**Formula**	**Concentration (% area)**
26,098	89	1-Hexadecyne	C_16_H_30_	0.93 **±** 0.11
26,306	88	Tetradecanoic acid, trimethylsilyl ester	C_17_H_36_O_2_Si	3.48 ± 0.10
28,996	80	9,12-Octadecadiynoic acid, trimethylsilyl ester	C_21_H_36_O_2_Si	25.19 ± 0.25
29,129	87	Linoleic acid trimethylsilyl ester	C_21_H_40_O_2_Si	2.65 ± 0.03
29,246	90	.alpha.-Linolenic acid, trimethylsilyl ester	C_21_H_38_O_2_Si	13.74 ± 0.12
29,314	84	Oleic acid, trimethylsilyl ester	C_21_H_42_O_2_Si	3.21 ± 0.09
29,637	83	Eicosanoic acid, trimethylsilyl ester	C_23_H_48_O_2_Si	17.14 ± 0.69
29,712	87	Arachidonic acid	C_20_H_32_O_2_	2.23 ± 0.04
32,240	88	11,14-Eicosadienoic acid, trimethylsilyl ester	C_23_H_44_O_2_Si	2.52 ± 0.04
32,363	82	cis-5,8,11-Eicosatrienoic acid, trimethylsilyl ester	C_23_H_42_O_2_Si	25.50 ± 0.35
34,669	82	Doconexent	C_22_H_32_O_2_	3.42 ± 0.17

The data shows in means followed by standard deviation (*n* = 2).

In control, we found a high number of organic compounds, namely Eicosanoic acid, trimethylsilyl ester (C_23_H_48_O_2_Si) accounted for 18.57 %. This compound was categorized as a fatty acid derivative compound often found in microalgae. Previous research by Arslan et al. (2023) reported that in *Spirulina platensis* Eicosanoic acid, 2,3-bis[(trimethylsilyl)oxy]propyl ester was found in the amount of 0.42 % at retention time (rt) 19,238. Furthermore, in the 5 % CO_2_ treatment, organic compounds were dominated by isophytol, acetate (C_23_H_48_O_2_Si) by 31.44 %. Isophytol acetate was a terpenoid alcohol compound with a molecular weight of 338.56. This compound was also found in *Lantana camara* Linn. extracts [38]. Another previous research has confirmed that isophytol compounds were obtained from the microalgae *Prorocentrum donghaiense* and *Heterosigma akashiwo* [39]*.*

Additionally, under 15 % CO_2_ condition, the dominant organic compound was cis-4,7,10,13,16,19-Docosahexaenoic acid, trimethylsilyl ester (C_25_H_40_O_2_Si) at 27.17 %. Previous research conducted by Fernandes and Cordeiro (2022) reported that the microalgae *Hemiselmis* cf. *andersenii* contained the compound 4,7,10,13,16,19-Docosahexaenoic acid around 1.68 mg/g. Docosahexaenoic acid (DHA – C22:6ω3) has established health benefits such as reducing the risk of cardiovascular disease, anti-inflammatory properties, and neuroprotective effects. It is important to understand the factors that trigger the production of these fatty acids in microalgae to expand their applications in the food and pharmaceutical sectors. DHA, a long-chain ω3-polyunsaturated fatty acid, is a crucial component of cell membranes. It is predominantly present in neuronal membranes and the outer segments of photoreceptors in the retina [40]. Besides, under the condition of 30 % CO_2_, the organic compound component cis-5,8,11-Eicosatrienoic acid, trimethylsilyl ester (C_23_H_42_O_2_Si) was obtained with a concentration of 25.50 % area. A previous study also revealed that eicosatrienoic acid compound was detected in at least 0.58 % of total fatty acids in euglenoid *Eutreptiella* sp. isolated from Korean coastal waters [41]. As mentioned by [42], 5,8,11-eicosatrienoic acid or mead acid is an n-9 polyunsaturated fatty acid (PUFA). This compound can be found in several microalgae species, such as *Chlorella* sp. S14 [43], *Cylindrotheca Closterium* [44], *Nannochloropsis oceanica* and *Phaeodactylum tricornutum* [45,46]. represented that omega-3 very long-chain polyunsaturated fatty acids (VLC-PUFAs), specifically eicosapentaenoic acid (EPA, C20:5Δ5,8,11,14,17) and docosahexaenoic acid (DHA, C22:6Δ4,7,10,13,16,19), are commonly utilized as dietary supplements because of their numerous health benefits.

### 3.5 Paramylon characterization

**Fig. 6:**
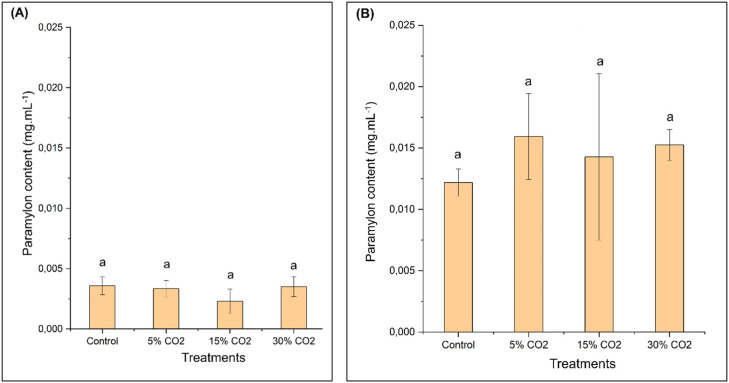
Paramylon content of *E. gracilis* at various CO₂ concentrations: (A) Day 0 of cultivation, (B) Day 6 of cultivation.

In this study, we extracted paramylon (β−1,3-glucan) and analyzed its morphological characteristics using scanning electron microscopy (SEM). The quantification results of paramylon of *E. gracilis* are presented in Fig. 6.

**Fig. 7:**
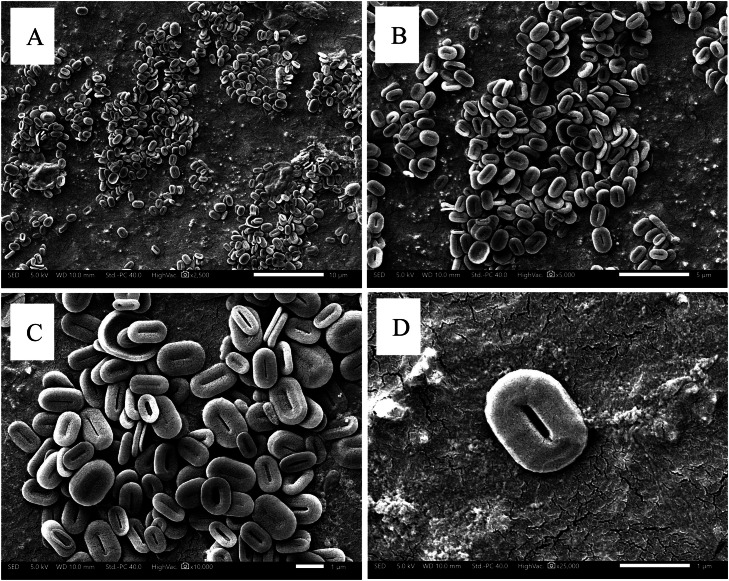
Photomicrograph of *E. gracilis* paramylon at various magnifications; A) 2.500x magnification. B) 5.000x magnification. C) 10.000x magnification. D) 25.000x magnification.

Based on the figure above, the paramylon content on day 0 showed no significant differences, as determined by One-way ANOVA. The paramylon concentration on day 0 ranged from 0.0006 to 0.001 g/L. Meanwhile, on day 6 of cultivation, the highest paramylon content was observed in the 5 % CO₂ treatment, reaching 0.0159 ± 0.0034 g/L. Statistical analysis using ANOVA, followed by a post hoc test with DMRT, indicated no significant differences between the treatment groups and the control. Furthermore, the morphological analysis results of *E. gracilis* paramylon are presented in Fig. 7.

The results of this study also support previous research by [47], which found that the paramylon content in *E. gracilis* increases most rapidly over time with longer cultivation under 5 % CO₂ conditions. Paramylon derived from *E. gracilis* is a primary assimilative product, and its content effectively reflects the physiological response of microalgal cells to environmental stimuli. Excessive CO₂ aeration concentrations can reduce paramylon accumulation, thus confirming that a 5 % CO₂ concentration is the most optimal condition for cultivating *E. gracilis*. However, when the CO₂ concentration reaches 20 %, the stimulatory effect on paramylon synthesis gradually decreases, following the same trend as changes in cell density [47,48].

The SEM image reveals the ultrastructural characteristics of paramylon granules in *E. gracilis*, highlighting their distinct morphology and size. The observed paramylon granules exhibit a disk-like (oval to ellipsoid) shape with a central hole. Furthermore, the size of paramylon granules varies, with diameters typically ranging from 0.5 to 2.5 µm (as indicated by the scale bar). This is supported by previous research conducted by Anraku et al. (2020) that the internal structure of paramylon particles (5 µm in diameter and 3 µm in thickness) was densely packed, with no detectable large pores or gaps.

Paramylon in *E. gracilis* serves as an energy reserve, particularly under unfavourable environmental conditions. Additionally, it functions as a dietary fibre, an immunomodulatory agent, and a precursor for pharmaceuticals and health supplements. A previous study reported that *E. gracilis* EOD-1 is known for its high production of paramylon, a type of β−1,3-glucan polymer. This compound has been recognized for its role as a dietary fibre and its potential benefits in alleviating metabolic syndrome, including obesity [49].

### 3.6 Gene expression of *E. gracilis* under 5 % CO_2_ and control

**Fig. 8:**
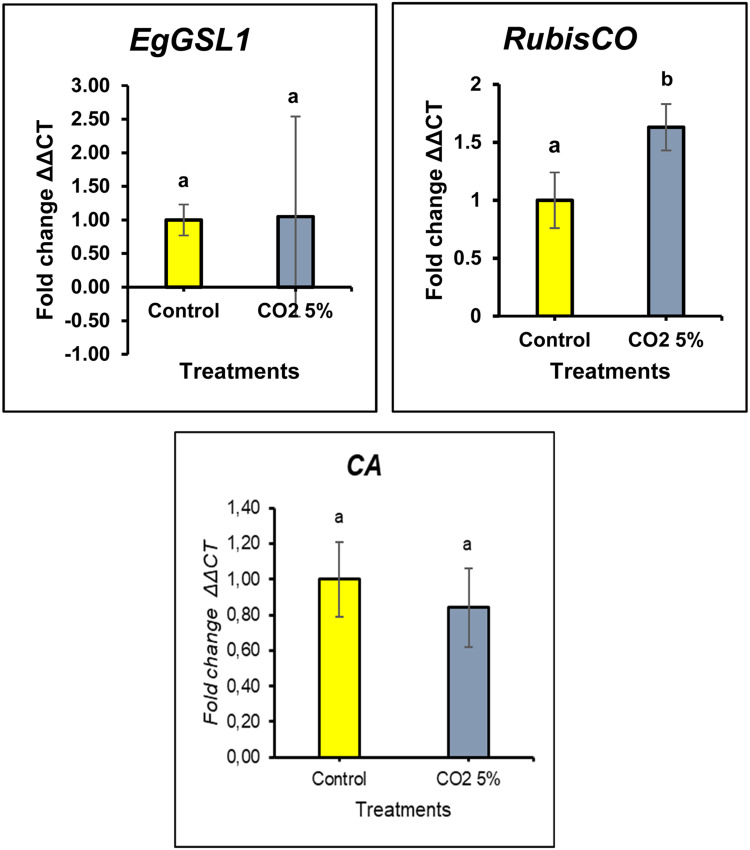
Comparison of *RubisCO, CA*, and *EgGSL1* gene expression levels in control and 5 % CO_2_ treatments.

In the gene expression analysis, we focused on the most effective treatment based on growth, biomass production, and paramylon content. Therefore, this analysis compared the optimal treatment, 5 % CO₂, with the control group (without CO₂ injection). Gene expression analysis was performed on several genes involved in the carbon fixation pathway, including Ribulose-1,5-bisphosphate carboxylase (*RubisCO*) and Carbonic anhydrase (*CA*), as well as a gene associated with paramylon biosynthesis, namely paramylon β−1,3 glucan synthase (*EgGSL1*). In this analysis, only the optimal CO₂ treatment (5 % CO₂) was tested and compared with the control group. The results of the gene expression analysis for *RubisCO, CA*, and *EgGSL1* are presented in Fig. 8.

The expression profiles of three key genes, *RubisCO* (Ribulose-1,5-bisphosphate carboxylase/oxygenase), *CA* (Carbonic Anhydrase), and *EgGSL1* (β−1,3-Glucan Synthase) were analyzed in *E. gracilis* cultured under ambient (control) and 5 % CO₂ supplementation. The results, presented in Fig. 8, demonstrate differential gene expression patterns in response to elevated CO₂ levels, reflecting physiological and metabolic adaptations.

Based on these results, the expression of the *EgGSL1* gene did not exhibit significant changes between the control condition and 5 % CO₂ treatment. This indicates that *EgGSL* is not directly influenced by elevated CO₂ levels. *EgGSL1* plays a crucial role in the biosynthesis of β−1,3-glucan [21]. Although the slight increase in *EgGSL1* expression was not statistically significant, it suggests that *EgGSL1* has a relatively minor role in responding to CO₂ exposure. The expression level of *EgGSL1* under 5 % CO₂ treatment showed a 1.05-fold increase compared to the control. The relatively stable expression of *EgGSL1* under 5 % CO₂ indicates that CO₂ supplementation does not significantly affect paramylon biosynthesis at the transcriptional level.

*RubisCO* is a pivotal enzyme in the Calvin-Benson cycle, catalyzing the fixation of CO₂ into organic compounds [50]. The expression of the *RubisCO* gene exhibited a statistically significant upregulation (*p* < 0.05) under 5 % CO₂ enrichment, indicating an adaptive response to enhanced carbon availability. This increased expression suggests that *E. gracilis* enhances its photosynthetic carbon assimilation capacity under elevated CO₂, aligning with previous studies that report enhanced growth rates and photosynthetic efficiency in microalgae under CO_2_ enrichment. The observed transcriptional regulation of *RubisCO* further supports its role in facilitating higher biomass accumulation, as evidenced in various algal species exposed to elevated CO₂ concentrations.

Carbonic anhydrase (*CA*) plays an important role in the interconversion of CO₂ and bicarbonate (HCO₃^−^), thus facilitating the transport and utilization of CO₂ [18]. In contrast to RubisCO, *CA* expression remained statistically unchanged (*p* > 0.05) between control and 5 % CO₂ conditions. This shows that the intracellular carbon conversion process is not significantly affected at the transcription rate by moderate CO₂ enrichment. A possible explanation is that *E. gracilis* maintains a regulated carbon concentration mechanism (CCM) that prevents excessive fluctuations in CO₂ utilization. In addition, the stability of *CA* expression under increased CO₂ availability may indicate that bicarbonate-dependent CO₂ transport is not a limiting factor under these conditions.

### 3.7 Evaluation of CO_2_ biofixation ability

**Fig. 9:**
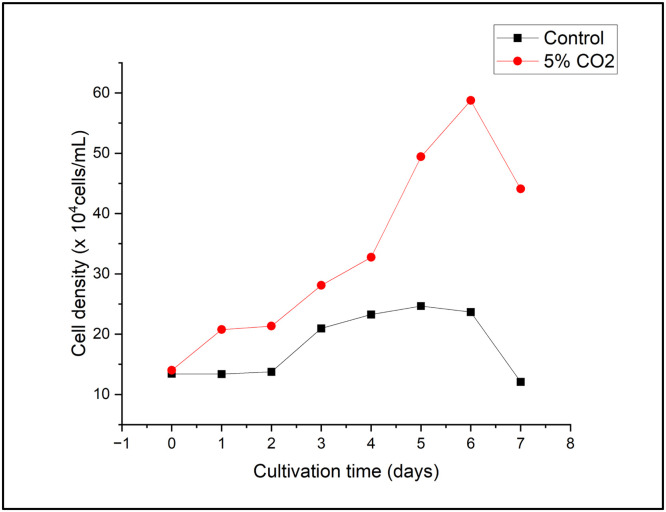
The cell density of *E. gracilis* after scaling up to 12 L cultivation.

**Fig. 10:**
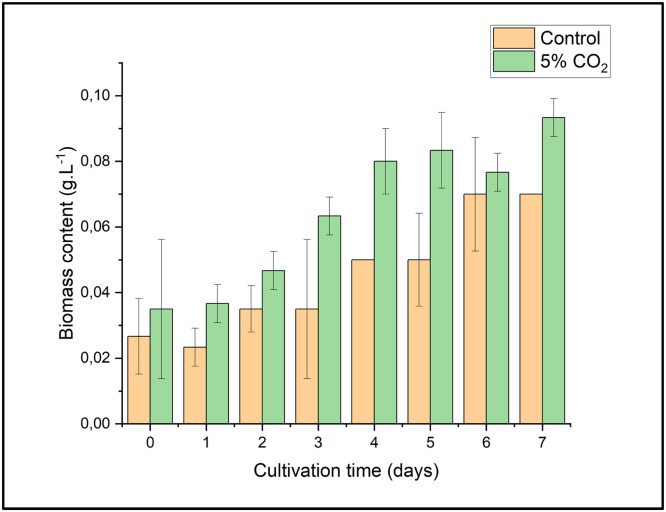
Biomass content of *E. gracilis* after scaling up to 12 L cultivation.

To evaluate the CO₂ absorption capacity of *E. gracilis*, we scaled up the cultivation to a 12-liter system using CM medium. Additionally, the growth parameters of *E. gracilis* were assessed, including dry biomass, cell density, specific growth rate (SGR), doubling time, and CO₂ capture rate. The growth curve of *E. gracilis*, based on cell density measurements, is presented in Fig. 9, while the dry biomass data is shown in Fig. 10.

The growth curve of *E. gracilis* on a pilot scale follows the same pattern as the results observed in laboratory-scale research (500 mL). Consistent with the previous growth curve, *E. gracilis* cultivated with a 5 % CO₂ gas injection exhibited a peak growth phase on the 6th day of cultivation. In contrast, by the 7th day, the cells had already entered the death phase.

A significant difference was also observed in the control group, where the cell count was considerably lower compared to the CO₂-injected cultures. In the control group, the lag phase occurred from day 0 to day 2, followed by the exponential phase from day 2 to day 3. The stationary phase was observed from day 4 to day 6.

Based on the biomass calculation results, *E. gracilis* cultivated with CO_2_ injection exhibited higher biomass production than the control group. Cultivation on a 12 L scale demonstrated that the dry biomass yield was consistent with biomass accumulation observed in laboratory-scale cultivation (500 mL). At this stage, an evaluation of the CO_2_ absorption rate was also conducted to assess the CO_2_ bio-fixation capability of *E. gracilis* by comparing the optimal cultivation condition (5 % CO_2_ injection) with the control group. Table 8 presents the highest dry biomass content, biomass productivity, specific growth rate, division per day, doubling time, and CO_2_ capture rate.

**Table 8:** Biomass content, biomass productivity, specific growth rate (SGR), doubling time, and CO_2_ capture of *E. gracilis*.

Treatments	Biomass content (g.L^−1^)	Biomass Increase ( %)	Biomass productivity (g.L^−1^.day^−1^)	Biomass productivity increase ( %)	Specific growth rate (µ.day^−1^)	Division per day (Dd)	Doubling time, td (day)	CO_2_ inlet (ppm)	CO_2_ outlet (ppm)	CO_2_ capture (ppm)	% CO_2_ capture	CO_2_ capture rate (g.L^−1^.day^−1^)
Control	0.07	-	0.066	-	0.138	0.199	5.028	1756	1400	356	20 %	0.006
5 % CO_2_	0.093	32.86 %	0.088	33.33 %	0.140	0.202	4.947	50,000	27,140	22,860	46 %	0.429

The data presented in Table 8 demonstrate the impact of CO₂ enrichment on *E. gracilis* growth and carbon capture. The findings indicate that increasing the CO₂ concentration to 5 % enhances biomass accumulation, productivity, and CO₂ capture efficiency, which are crucial factors in optimizing microalgal cultivation for biotechnological applications.

These results indicate a significant improvement in biomass content when *E. gracilis* was cultured under 5 % CO₂ supplementation. Compared to the control condition (0.07 g/L), the biomass content under 5 % CO₂ increased to 0.093 g/L, reflecting a 32.86 % enhancement. This suggests that elevated CO₂ concentrations promote higher biomass accumulation, likely due to enhanced photosynthetic efficiency and improved carbon fixation [51].

Similarly, biomass productivity exhibited a notable increase from 0.066 g/L/day in the control to 0.088 g/L.day under 5 % CO₂, representing a 33.33 % improvement. This demonstrates that higher CO₂ availability optimizes metabolic activities and cell growth, leading to better biomass yield. The findings align with previous studies highlighting the role of CO₂ enrichment in stimulating microalgal growth. However, despite the positive effects observed at 5 % CO₂, further optimization may be necessary to balance growth rates, metabolic efficiency, and CO₂ utilization for large-scale applications. Future research could explore different CO₂ concentrations, aeration rates, and light conditions to maximize biomass production. This finding aligns with previous studies showing that microalgae generally benefit from CO₂ supplementation, as it provides an essential carbon source for photosynthesis and organic carbon metabolism [47,52]. *E. gracilis* is known for its ability to assimilate CO₂ efficiently and store energy in the form of paramylon, a β−1,3-glucan polymer that serves as a crucial metabolic reserve [47].

The specific growth rate (SGR) increased slightly from 0.138 μ·day⁻¹ (control) to 0.140 μ·day⁻¹ under 5 % CO₂ conditions, with the division rate improving from 0.199 divisions per day to 0.202 divisions per day. Although the increase in growth rate is minimal, the doubling time (td) decreased from 5.028 days to 4.947 days, suggesting that *E. gracilis* cells divide slightly faster in the presence of elevated CO₂. Similar observations have been reported in other microalgal species, where increased CO₂ enhances photosynthetic efficiency and reduces the energy required for carbon fixation [53], the relatively small improvement in growth rate suggests that *E. gracilis* may already be operating near its optimal carbon assimilation capacity under ambient CO₂ levels.

A significant difference was observed in CO₂ capture between treatments. Under control conditions, the CO₂ inlet was 1756 ppm, with an outlet concentration of 1400 ppm, leading to a CO₂ capture of 356 ppm (20 % efficiency). In contrast, at 5 % CO₂, the inlet concentration increased to 50,000 ppm, with an outlet of 27,140 ppm, resulting in a CO₂ capture of 22,860 ppm—an efficiency of 46 %. The CO₂ capture rate also increased dramatically from 0.006 g·L⁻¹·day⁻¹ (control) to 0.429 g·L⁻¹·day⁻¹ under 5 % CO₂, which corresponds to an approximate 7150 % improvement. These findings demonstrate that *E. gracilis* is highly effective in CO₂ sequestration, particularly at elevated CO₂ concentrations. Previous studies have reported that microalgae such as *Chlorella* and *Scenedesmus* exhibit similar increases in CO₂ capture when exposed to high CO₂ levels, making them promising candidates for bioremediation and carbon capture technologies [54,55].

While increased CO₂ availability enhances biomass production and carbon capture, excessive CO_2_ can sometimes lead to cellular stress due to acidification of the culture medium, affecting enzyme activity and metabolism [56,57]. However, *E. gracilis* is known for its adaptability to fluctuating CO₂ levels, utilizing both photosynthesis and mixotrophic metabolism to optimize growth under different environmental conditions [52]. The stability of growth parameters in this study suggests that 5 % CO₂ does not exert a toxic effect on *E. gracilis*, making it a suitable candidate for large-scale cultivation in CO₂-rich environments, such as industrial flue gas bioremediation.

## 4 Discussion

The growth performance, metabolic response, and CO₂ absorption efficiency of *E. gracilis* at different CO₂ concentrations were assessed in this study. The results demonstrated that biomass accumulation and lipid production were all markedly enhanced in an environment with 5 % CO₂. The molecular mechanisms behind carbon fixation and energy storage in *E. gracilis* are also highlighted by gene expression analysis, underscoring the organism's potential as a sustainable biological resource for CO_2_ mitigation and bioproduct development. This study indicates that *E. gracilis* can tolerate various CO_2_ levels without significant inhibition within six days of cultivation. Previous research by Xin et al. (2023) that when CO_2_ concentration exceeds 15 %, microalgae growth is generally inhibited. The inhibition rate and growth delay period increased as the CO_2_ concentration increased, while the stimulating effect of CO_2_ domestication also increased.

However, *E. gracilis* cultivated at 15 % CO_2_ produced the highest DHA (Docosahexaenoic acid) up to 27.17 %. DHA is an omega-3 fatty acid important for cardiovascular and neurological health [58]. The biomass and lipid content in this treatment was not as high as when *E. gracilis* was cultivated at 5 % CO_2_, but at least in this condition, they were able to survive and did not experience a drastic decrease in productivity. In addition, *E. gracilis* cultivated under 30 % CO₂ showed some interesting metabolic changes, even though its growth was not optimal. The growth and biomass were not optimal and were close to the control, indicating stress or an inability to adapt to high CO₂ levels without domestication. Specifically, *E. gracilis* under these conditions was able to produce the highest amount of eicosatrienoic acid, reaching 25.50 %. This compound is produced when there is a lack of essential fatty acids, indicating metabolic stress. According to [42] explained that mead acid (MA, 5,8,11-eicosatrienoic acid) is a type of n-9 polyunsaturated fatty acid (PUFA) that serves as an indicator of essential fatty acid deficiency.

At moderate CO_2_ levels (*e.g.*, 5–15 %), dissolved CO_2_ equilibrates with bicarbonate, slightly decreasing the external pH, which is balanced by the cell’s buffering system [59,60]. Under these conditions, *E. gracilis* maintains an active carbon concentrating mechanism (CCM) to capture inorganic carbon efficiently, directing carbon dioxide primarily toward paramylon synthesis as a carbohydrate storage compound [61].

However, at very high CO₂ levels (*e.g.*, 30 %), excessive dissolved CO₂ can significantly reduce external and intracellular pH, leading to partial inactivation of the CCM [62]. When CCM activity declines, the cells experience metabolic stress and redirect carbon flux away from carbohydrate accumulation toward fatty acid synthesis as an alternative storage pathway. This shift explains the increase in specific fatty acids, such as DHA and mead acid, under high CO₂ stress conditions, while overall biomass growth remains low [63].

The 5 % CO₂ treatment produced the highest biomass productivity and cell density, which reached 0.407 ± 0.099 g/L on day 6 (Fig. 2). This rise suggests that *E. gracilis* effectively absorbed CO₂ as a carbon source to promote cell division. The enhanced growth rate is consistent with earlier research that found microalgal metabolism and photosynthetic efficiency are enhanced by moderate CO₂ concentrations (5–10 %) [[30], [47]]. Beyond 5 % CO₂, *E. gracilis's* growth drastically declined, despite demonstrating resistance to higher CO₂ levels (15 and 30 %). According to the previous research, excessive CO₂ caused growth inhibition due to intracellular acidification and metabolic imbalance [14,31]. These findings imply that CO₂ saturation or acidification may have caused physiological stress. Similar trends have been previously reported in other microalgal species, such as *Chlorella* and *Scenedesmus*, where optimal CO₂ concentrations (5–10 %) enhance growth, but further increases reduce photosynthetic efficiency [11,13].

Increased biomass productivity under 5 % CO₂ is likely due to increased carbon assimilation through the Calvin cycle and alternative metabolic pathways. The upregulation of RuBisCO (Ribulose-1,5-bisphosphate carboxylase/oxygenase) under 5 % CO₂ supports this hypothesis, as RuBisCO plays an important role in CO₂ fixation [50]. In addition, the expression of carbonic anhydrase (*CA*), which facilitates the conversion of CO₂ to bicarbonate (HCO₃^−^), remained stable, suggesting that *E. gracilis* maintains a well-regulated carbon concentration mechanism even under increased CO₂ availability [18].

Lipid synthesis in *E. gracilis* was markedly increased under 5 % CO₂, attaining 0.189 ± 0.025 g/L by day 6 (Fig. 4). The noted increase aligns with the discovery that moderate CO₂ levels enhance lipid accumulation in microalgae by redirecting carbon flux towards fatty acid biosynthesis [64,65]. GC–MS analysis identified isophytol acetate (31.44 %) as the predominant compound under 5 % CO₂ conditions (Table 5). This compound, a precursor for chlorophyll biosynthesis and biofuel applications, has also been documented in other microalgae, including *Prorocentrum donghaiense* and *Heterosigma akashiwo* [39]. Docosahexaenoic acid (DHA, C22:6ω3) was the predominant compound at 15 % CO₂ (27.17 %), underscoring the impact of CO₂ availability on lipid composition [66]. DHA, a long-chain omega-3 polyunsaturated fatty acid (PUFA), is esteemed for its anti-inflammatory and neuroprotective attributes, positioning *E. gracilis* as a viable candidate for nutraceutical applications [40]. At 30 % CO₂, cis-5,8,11-Eicosatrienoic acid (C_23_H_42_O_2_Si, 25.50 %) is the dominant fatty acid. This compound, also known as Mead's acid, is usually synthesized under essential fatty acid deficiency and is found in species such as *Nannochloropsis oceanica* and *Phaeodactylum tricornutum* [42,45]. The increase in Mead's acid suggests that *E. gracilis* may have adapted its lipid metabolism to cope with CO₂-induced stress.

CO₂ enrichment enhances lipid accumulation by diverting photosynthetic carbon towards triacylglycerol (TAG) biosynthesis. The metabolic transition is enabled by enhanced NADPH availability, promoting fatty acid elongation and desaturation [37]. Upregulation of essential lipid biosynthetic enzymes, including acetyl-CoA carboxylase (ACC), was observed in a comparable study involving CO₂-suppressed microalgae [64]. The findings indicate that optimizing CO₂ levels can modulate lipid biosynthesis in *E. gracilis,* presenting potential applications for biofuel production and the extraction of high-value lipids.

Paramylon (β−1,3-glucan) acts as an energy reserve in *E. gracilis* and plays an important role in stress tolerance. The highest paramylon content (0.0159 ± 0.0034 g/L) was observed under 5 % CO₂ treatment, consistent with studies showing that carbon flux is diverted to storage metabolism under excess CO₂ [21]. SEM analysis revealed that paramylon granules exhibited an oval to ellipsoid morphology with diameters ranging from 0.5 to 2.5 μm (Fig. 9). These structural characteristics are in line with previous findings that the physicochemical properties of paramylon affect its functionality in biomedical and biopolymer applications [49].

*EgGSL1* (β−1,3-glucan synthase) demonstrated a 1.05-fold increase in expression under 5 % CO₂; however, this change was not statistically significant. This indicates that the biosynthesis of paramylon in *E. gracilis* is not directly influenced by CO₂ availability, but is instead governed by nutrient equilibrium and environmental stress [21].

Although paramylon content increased under elevated CO₂ conditions, the expression of *EgGSL1* transcripts did not exhibit a corresponding change. This apparent discrepancy may be attributed to several regulatory mechanisms. The regulation of paramylon biosynthesis may primarily occur at post-transcriptional or post-translational levels, such as through alterations in translation efficiency, enzyme activation, or protein stability, where transcript abundance does not necessarily reflect enzyme activity or final product accumulation [67,68].

In addition, shifts in carbon partitioning and precursor availability, such as UDP-glucose, can indeed redirect metabolic flux toward paramylon synthesis in *E. gracilis* without necessitating changes in *EgGSL1* transcript levels. According to a previous study reported by Tanaka et al. (2017), *EgGSL1* does not show a significant impact on paramylon levels when knocked down, indicating that changes in *EgGSL1* transcript levels are not necessary for increased paramylon synthesis [21].

RuBisCO expression is markedly upregulated in the presence of 5 % CO₂, underscoring its function in improved carbon fixation and biomass accumulation [50]. The expression of carbonic anhydrase remained constant, indicating that bicarbonate conversion is not a limiting factor under these conditions [18]. The results underscore the metabolic adaptability of *E. gracilis* to variations in CO₂, positioning it as a robust candidate for carbon sequestration and bioproduct innovation.

Pilot-scale cultivation using a 12 L setup demonstrated that CO₂ absorption was higher under the 5 % CO₂ treatment, reaching 0.429 g·L⁻¹·day⁻¹, compared to just 0.006 g·L⁻¹·day⁻¹ in the control. In low-CO_2_ environments, the CO_2_ uptake ability of microalgae is constrained by carbon scarcity, whereas the 5 % CO_2_ condition provides an adequate supply without inducing acidification stress, thereby enhancing biofixation efficiency. This efficiency exceeds traditional CO₂ biofixation systems, establishing *E. gracilis* as a promising candidate for carbon-negative bioremediation and biotechnology [54,55]. Microalgae-based CO₂ sequestration presents a sustainable alternative to conventional carbon capture technologies, yielding bioproducts including biofuels, nutraceuticals, and biomaterials [7].

Furthermore, CO₂ is an essential carbon source for *E. gracilis*, especially when growing autotrophically. In the absence of CO₂, carbon availability is limited, restricting the synthesis of important biomolecules such as proteins, lipids, and carbohydrates. At low CO_2_ levels (0.04 %; v/v), carbon limitation occurs, leading to minimal accumulation of triacylglycerol (TAG) and starch in the microalgae *Chlamydomonas reinhardtii*. However, at high CO_2_ concentrations (5 %; v/v), *C. reinhardtii* produces the highest amount of biofuel precursors, which are subsequently transesterified into fatty acid methyl esters [69]. A previous study reported that elevated CO_2_ levels enhance photosynthetic carbon assimilation, leading to increased production of photosynthetic metabolites such as paramylon in *E. gracilis* [47]. On the other hand, at 5 % CO₂, there is a sufficient carbon supply, allowing for enhanced biomass production and faster growth. A previous report by [70] explained that algae utilize CO_2_ in the process of photosynthesis, converting it into organic compounds that form the basis of their biomass. This process is essential for their growth and energy production. In addition, other research reported by [71] showed that under high CO_2_ conditions 3 % v/v (30 000 ppm), *Chlorella saccharophila* show increased accumulation of lipids, proteins, and carbohydrates, which are essential for biofuel production.

## 5 Conclusion

This study confirms *E. gracilis* as a promising candidate for CO₂ capture and biomass production. The 5 % CO₂ treatment showed optimal performance, with the highest biomass (0.407 ± 0.099 g/L), lipid yield (0.189 ± 0.025 g/L), and dominant isophytol acetate content (31.44 %), supporting its potential in biofuel and pharmaceutical applications. Control conditions maintained stable growth and may serve as a baseline for low-input cultivation. At 15 % CO₂, the highest DHA content (27.17 %) was observed, suggesting potential for nutraceutical use. Although growth was limited at 30 % CO₂, the presence of stress-related metabolites like mead acid highlights its adaptive metabolic responses. Pilot-scale cultivation confirmed high CO₂ capture rate (0.429 g.L⁻¹.day⁻¹), reinforcing the scalability of *E. gracilis* for bioremediation. Further work on strain improvement and large-scale system optimization is needed to enhance commercial viability.

## CRediT authorship contribution statement

**Tia Erfianti:** Writing – review & editing, Writing – original draft, Methodology, Investigation, Formal analysis, Data curation, Conceptualization. **Brilian Ryan Sadewo:** Writing – review & editing, Writing – original draft, Visualization, Validation, Software, Formal analysis. **Nugroho Dewayanto:** Writing – review & editing, Writing – original draft, Supervision, Resources, Project administration, Methodology. **Adib Fakhruddin Yusuf:** Data curation, Formal analysis, Methodology, Supervision, Visualization, Writing – original draft, Writing – review & editing. **Dedy Kurnianto:** Validation, Writing – original draft, Writing – review & editing. **Deviko Mardyansah:** Methodology, Software, Validation, Visualization. **Ikhnu Prawestri Wardani:** Methodology, Validation, Visualization. **Budi Setiadi Daryono:** Supervision, Validation, Writing – original draft, Writing – review & editing. **Eko Agus Suyono:** Writing – review & editing, Writing – original draft, Visualization, Validation, Supervision, Investigation, Conceptualization. **Marcos:** Writing – review & editing, Writing – original draft, Validation, Supervision. **Arief Budiman:** Writing – review & editing, Writing – original draft, Validation, Supervision, Resources, Funding acquisition.

## Declaration of competing interest

The authors declare the following financial interests/personal relationships which may be considered as potential competing interests:

Arief budiman reports financial support was provided by LPDP - Indonesia Endowment Fund for Education Agency. If there are other authors, they declare that they have no known competing financial interests or personal relationships that could have appeared to influence the work reported in this paper.

## Data Availability

Data will be made available on request.

## Appendix Supplementary materials

Image, application 1

Image, application 2
