# Supplementary material for: Enhanced CO2 sequestration and paramylon production in acid-tolerant Euglena gracilis: Growth optimization and metabolic response under varying CO2 concentrations
Source: Biotechnol Rep (Amst). 2025 Oct 30;48:e00935. doi: 10.1016/j.btre.2025.e00935 (PMC12663494; doi:10.1016/j.btre.2025.e00935)
Supplement: Supplementary file 1 [file mmc1.pdf]

===

Acq. Operator : SYSTEM Seq. Line : 2
Acq. Instrument : GC 7890B Location : 141
Injection Date : 1/11/2023 11:47:07 AM Inj : 1
Inj Volume : 1 µl
Acq. Method : C:\Chem32\1\Data\Std FAME Mix 37 Supelco 110123 HP88 2023-01-11 10-48-57 \Methylester HP 88 Mix 37 Cal 110123.M
Last changed : 1/11/2023 10:48:58 AM by SYSTEM
Analysis Method : C:\Chem32\1\Data\Std FAME Mix 37 Supelco 110123 HP88 2023-01-11 10-48-57 \Methylester HP 88 Mix 37 Cal 110123.M (Sequence Method)
Last changed : 1/16/2023 8:17:16 AM by SYSTEM (modified after loading)
Method Info : asam lemak campuran

Additional Info : Peak(s) manually integrated

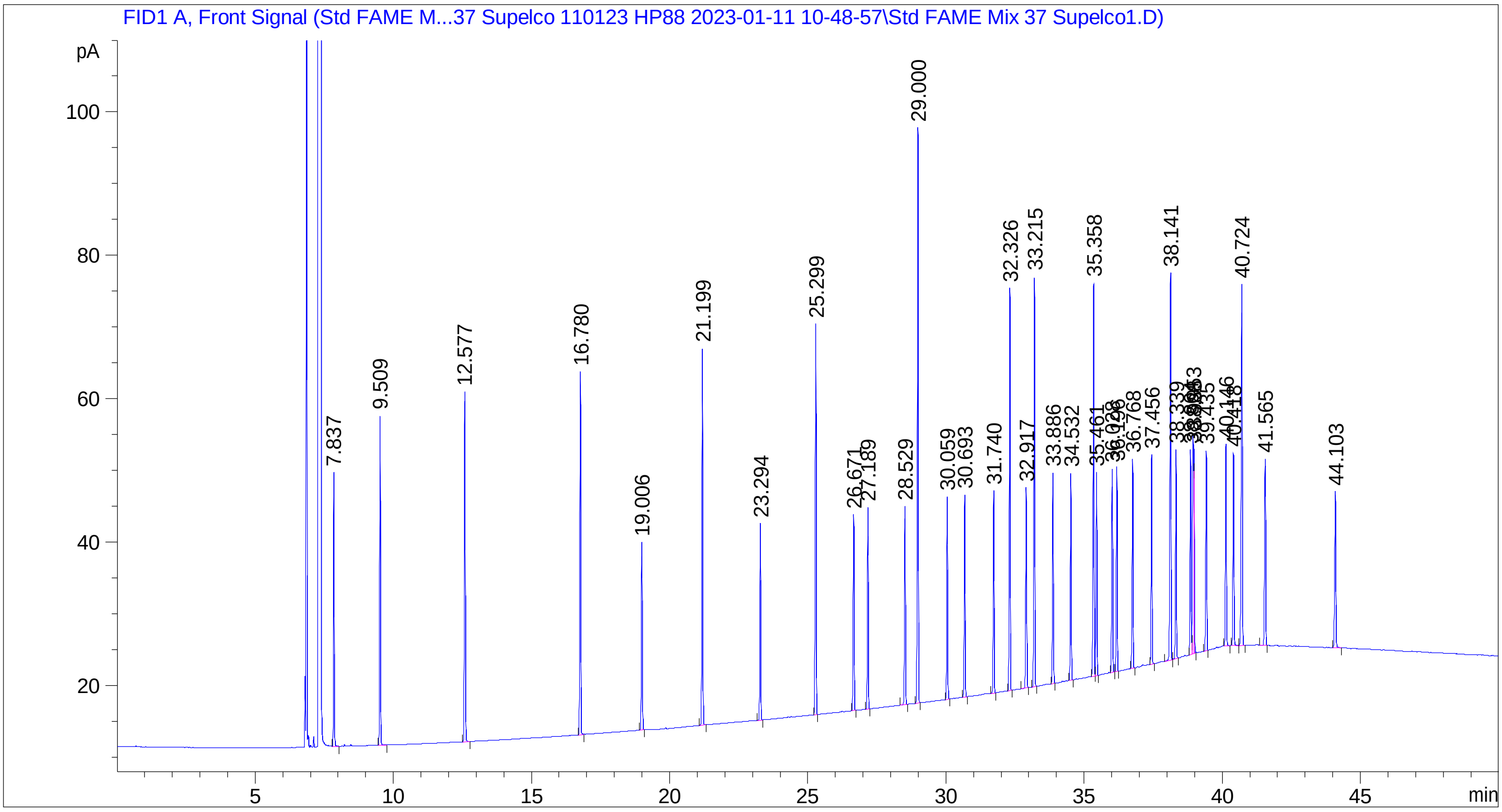

===

Area Percent Report

===

Sorted By : Signal
Calib. Data Modified : Thursday, January 12, 2023 11:21:05 AM
Multiplier : 1.0000
Dilution : 1.0000
Do not use Multiplier & Dilution Factor with ISTDs

Signal 1: FID1 A, Front Signal

Peak #	RetTime [min]	Type	Width [min]	Area [pA*s]	Area %	Name
1	7.837	BB	0.0303	73.23218	2.24854	Methyl butyrate
2	9.509	BB	0.0341	99.03255	3.04072	Methyl hexanoate

Peak #	RetTime [min]	Type	Width [min]	Area [pA*s]	Area %	Name
3	12.577	BB	0.0362	114.05300	3.50191	Methyl octanoate
4	16.780	BB	0.0370	121.57961	3.73301	Methyl decanoate
5	19.006	BB	0.0367	62.17271	1.90897	Methyl undecanoate
6	21.199	BB	0.0382	127.44135	3.91299	Methyl laurate
7	23.294	BB	0.0364	64.71177	1.98693	Methyl tridecanoate
8	25.299	BB	0.0370	131.06389	4.02422	Methyl myristate
9	26.671	BB	0.0367	65.15081	2.00041	Methyl myristoleate
10	27.189	BB	0.0375	66.55966	2.04366	Methyl pentadecanoate
11	28.529	BB	0.0377	65.86231	2.02225	Methyl cis-10 pentadecenoate
12	29.000	BB	0.0390	199.87010	6.13686	Methyl palmitate
13	30.059	BB	0.0374	66.66926	2.04703	Methyl palmitoleate
14	30.693	BB	0.0380	67.71311	2.07908	Methyl heptadecanoate
15	31.740	BB	0.0369	67.43941	2.07068	Methyl cis-10 heptadecenoate
16	32.326	BB	0.0374	136.87917	4.20277	Methyl stearate
17	32.917	BB	0.0374	68.18909	2.09369	Methyl trans-9 eladiate
18	33.215	BB	0.0370	137.40620	4.21895	Methyl cis-9 oleate
19	33.886	BB	0.0361	68.36799	2.09919	Methyl linolelaidate
20	34.532	BB	0.0380	69.47567	2.13320	Methyl linoleate
21	35.358	BV	0.0385	138.97803	4.26721	Methyl arachidiate
22	35.461	VB	0.0379	68.02615	2.08869	Methyl-gamma-linolenate
23	36.028	BB	0.0381	68.52789	2.10410	Methyl cis-11-eicosanoate
24	36.196	BB	0.0378	68.17764	2.09334	Methyl linolenate
25	36.768	BB	0.0369	69.88506	2.14577	Methyl heneicosanoate
26	37.456	BB	0.0375	68.82294	2.11316	Methyl cis-11,14-eicosadienoate
27	38.141	BB	0.0418	142.43785	4.37345	Methyl docosanoate
28	38.339	BB	0.0366	69.13564	2.12276	Methyl cis-8,11,14-eicosatrienoate
29	38.864	BV	0.0365	67.46794	2.07155	Methyl erucate
30	38.953	VV	0.0386	77.55765	2.38135	Methyl cis-11,14,17-eicosatrienoate
31	38.988	VB	0.0315	60.15724	1.84708	Methyl tricosanoate
32	39.435	BB	0.0406	71.08750	2.18269	Methyl cis-5,8,11,14-eicosatetraenoate
33	40.146	BB	0.0385	68.77222	2.11160	Methyl cis-13,16-docosadienoate
34	40.418	BB	0.0389	66.57098	2.04401	Methyl lignocerate
35	40.724	BB	0.0409	142.25639	4.36787	Methyl cis-5,8,11,14,17-eicosapentaenoate
36	41.565	BB	0.0425	70.35597	2.16023	Methyl nervonate
37	44.103	BB	0.0471	65.79219	2.02010	Methyl cis-4,7,10,13,16,19-docosahexaenoate

Totals : 3256.87914

1 Warnings or Errors :

Warning : Calibration warnings (see calibration table listing)

==

*** End of Report ***
