# Supplementary material for: Enhanced CO2 sequestration and paramylon production in acid-tolerant Euglena gracilis: Growth optimization and metabolic response under varying CO2 concentrations
Source: Biotechnol Rep (Amst). 2025 Oct 30;48:e00935. doi: 10.1016/j.btre.2025.e00935 (PMC12663494; doi:10.1016/j.btre.2025.e00935)
Supplement: Supplementary file 2 [file mmc2.pdf]

Supplementary Figures

Figure S1. PCA plot of fatty acid profiles in *Euglena gracilis* under different CO_2 concentrations.

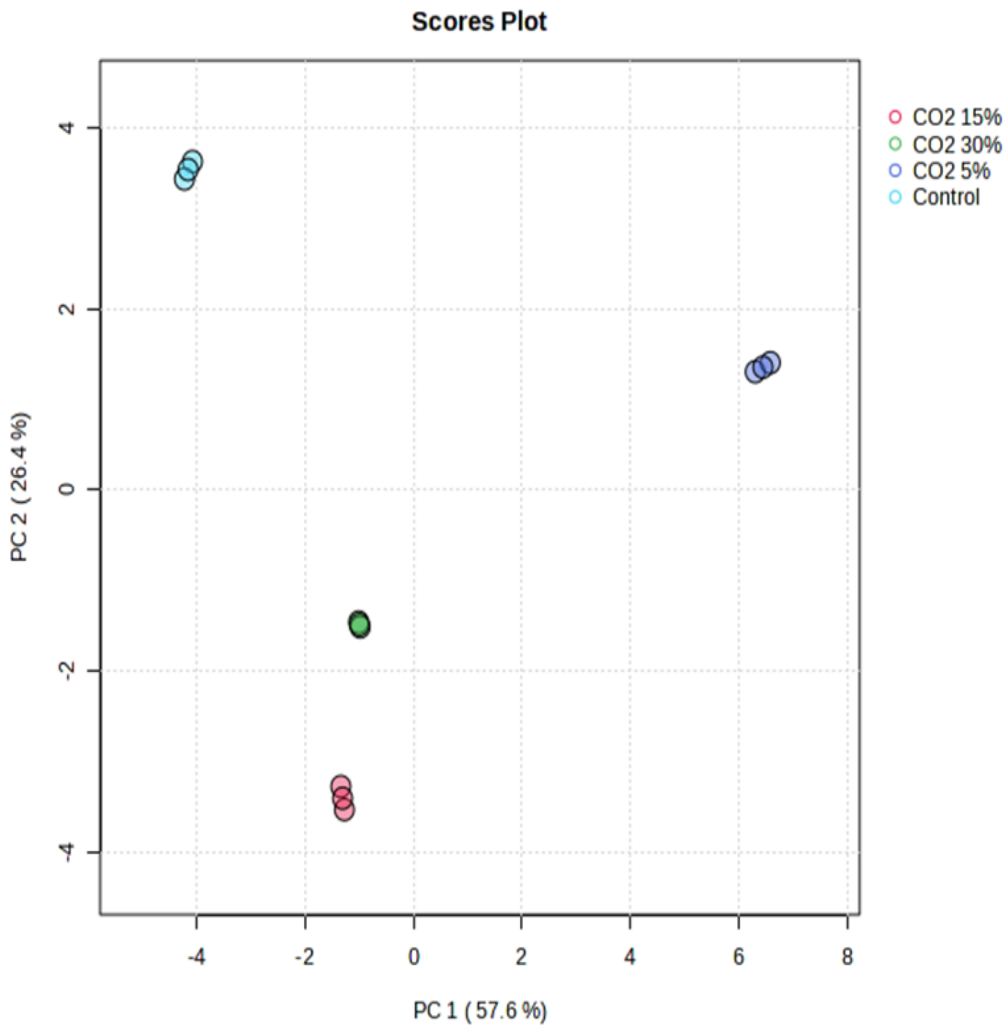

Figure S2. VIP scores of compounds detected in *Euglena gracilis* based on GC-MS analysis.

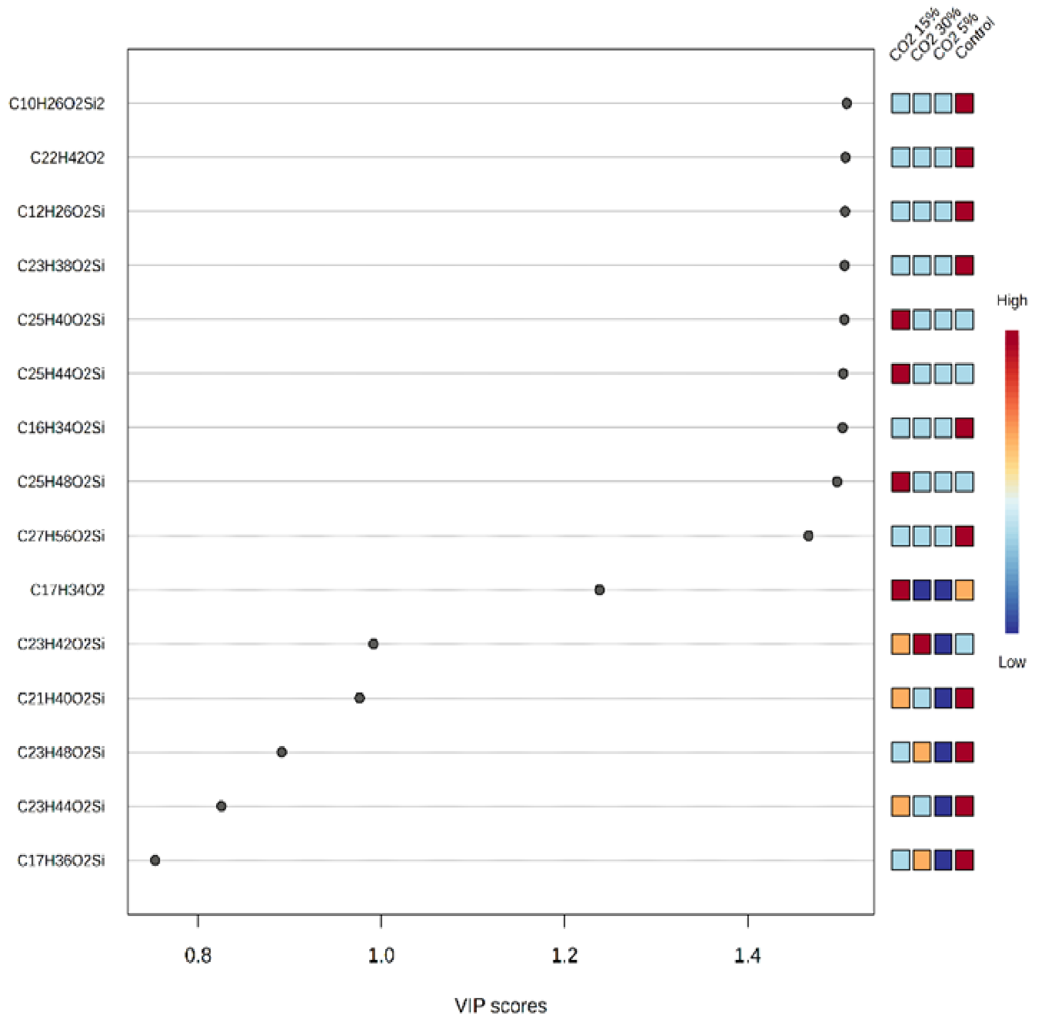

CO2 15%
CO2 30%
CO2 5%
Control
C10H26O2Si2
C22H42O2
C12H26O2Si
C23H38O2Si
C25H40O2Si
C25H44O2Si
C16H34O2Si
C25H48O2Si
C27H56O2Si
C17H34O2
C23H42O2Si
C21H40O2Si
C23H48O2Si
C23H44O2Si
C17H36O2Si
High
Low
0.8
1.0
1.2
1.4
VIP scores
